# The Coloring Mechanism of Opaque Glazes from the Changsha Kiln of the Tang Dynasty

**DOI:** 10.3390/ma17235803

**Published:** 2024-11-27

**Authors:** Lala Jia, Maolin Zhang, Yongqiang Yu, Zihan Li, Yongbin Yu

**Affiliations:** 1 Ancient Ceramic Research Center, Jingdezhen Ceramic University, Jingdezhen 333403, China; lys-la@hotmail.com (L.J.); zhangmaolin@jcu.edu.cn (M.Z.); wsyyqnl@163.com (Y.Y.); 2 Jiangxi Provincial Institute of Cultural Relics and Archaeology, Nanchang 330095, China; 3 School of Archaeology, Museology, Chinese Civilization, Nanjing University, Suzhou 215009, China; zihan.li@nju.edu.cn; 4 School of Archaeology, University of Oxford, Oxford OX1 3TG, UK; 5 National Centre for Archaeology, National Cultural Heritage Administration, Beijing 100013, China

**Keywords:** Changsha Kiln, opaque glaze, coloring mechanism, liquid–liquid phase separation

## Abstract

The Changsha Kiln of the Tang Dynasty was a major site for celadon production, yet the mechanisms behind the unique coloring of its opaque glazes remain inadequately explored. Prior research has largely focused on the general composition of these glazes, with limited understanding of the specific processes contributing to their distinct visual characteristics. This gap in knowledge hinders efforts to accurately replicate these historical glazes and fully appreciate their technological significance. In this study, we analyze the chemical composition and microstructure of opaque glaze specimens from the Changsha Kiln using advanced techniques such as EDXRF, SEM-EDS, and ultra-deep field microscopy. Our findings reveal that the opaque glazes are high-calcium compositions where phase separation and the presence of coloring ions like Cu^2+^, Cu^+^, and Fe^3+^ play critical roles in determining the glaze’s color. The interplay between chemical coloring and phase separation processes is shown to produce the distinct blue-green and creamy white hues observed in the glazes. These results provide a deeper understanding of the coloring mechanisms in Changsha Kiln glazes, contributing to the broader field of ceramic research and aiding in the preservation and accurate reproduction of these historic artifacts.

## 1. Introduction

The Changsha Kiln was a prominent site for celadon production during the Tang and Five Dynasties Period, gaining renown for its porcelain both domestically and internationally [1]. This kiln significantly contributed to ceramic technology, particularly in the mass production of underglaze porcelain and the development of the first high-melting temperature copper-red glaze and pigment.

In 1999, archaeological excavations by the Changsha Institute of Cultural Relics and Archaeology unearthed a 300-square-meter area in Lan’anzui, within the western part of the Changsha Kiln site. This excavation primarily revealed porcelain tiles and saggar piles, along with a substantial collection of celadon porcelain and celadon glaze with brown porcelain. Notably, a variety of opaque glaze porcelain in green, red, and blue colors was discovered [2].

Previous studies have provided insights into the composition and appearance of these opaque glazes. Zhang Fukang et al. suggested that the milky appearance of the opaque glazes is due to the presence of small amounts of SnO_2_, As_2_O_3_, and P_2_O_5_ [3]. Chen Xianqiu identified the glaze as phase-separated, with droplet sizes ranging from 185 nm to 405 nm, contributing to its creamy white appearance [4]. Following their analysis of a copper-red porcelain specimen from the Changsha Kiln, Li Yuanqiu et al. concluded that it was glazed prior to being painted and that the significant amount of liquid–liquid phase separation led to the milky appearance of the glaze [5].

Despite these findings, there remains a lack of comprehensive scientific understanding of the mechanisms underlying the formation and coloring of the opaque glazes from the Changsha Kiln. Addressing this gap, the present study focuses on the detailed analysis of opaque glaze porcelain specimens from the Tang Dynasty, excavated in 1999. We employed energy dispersive X-ray fluorescence (EDXRF, EDAX, Shanghai, China), scanning electron microscopy with energy dispersive spectroscopy (SEM-EDS- JEOL Corporation, Shanghai, China), and an ultra-deep field microscope to analyze the chemical composition and microstructure of these specimens.

Our investigation aimed to elucidate the mechanisms of chemical coloring, the formation of phase separation structures, and the physical coloring processes within the glaze. By exploring the intrinsic relationships between the glaze formulation, structure, and resulting coloration, this study seeks to provide a scientific basis for a deeper understanding of the technological and aesthetic connotations of opaque glaze porcelain from the Changsha Kiln.

## 2. Experimental Specimens and Methodology

### 2.1. Experimental Specimens

**Figure 1:**
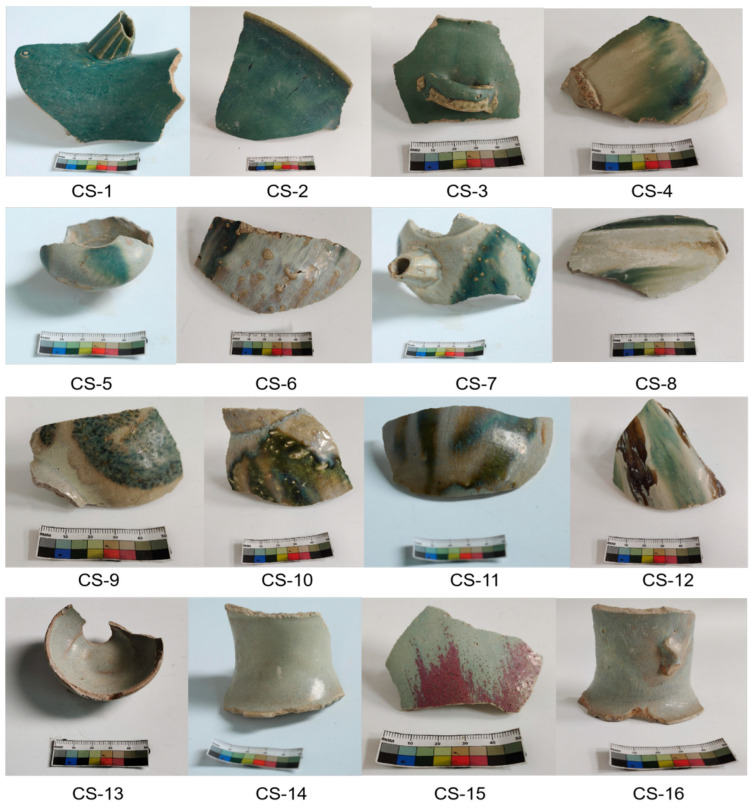
Sample of milky turbidness glaze of Changsha Kiln.

The Changsha Institute of Cultural Relics and Archaeology provided 16 opaque glaze fragments for analysis in this study. These fragments, excavated from the Lan’anzui site, covered all possible colors, are labelled CS-1 to CS-16 and are presented in Figure 1. Each specimen features a thin glaze layer with significant opacification. The visual characteristics of the specimens are briefly described and listed in Table 1. In Table 1, glaze thickness was determined using a VHX-6000 ultra-deep field microscope (Keyence, Shanghai, China). Cross-sectional images of the glazes were taken to measure the thickness at multiple points, ensuring accuracy. The mean value from five measurements per sample was recorded. A representative sample, CS-1, exhibited a glaze thickness of 188.2 μm.

### 2.2. Experimental Method

The chemical composition of the glaze specimens was analyzed using an Eagle-III energy dispersive X-ray fluorescence (EDXRF) spectrometer, manufactured by EDAX USA (Shanghai, China). This instrument is equipped with a side-window rhodium target, a 50 mW X-ray tube, a down-irradiation type, and an incident X-ray spot diameter of 300 μm, coupled with an Si(Li) detector. During the test, the X-ray tube operated at a pressure of 50 kV and a current of 200 μA, with a vacuum light path and a dead time of approximately 25%. To ensure accuracy and comparability in this nondestructive testing, a series of standard reference materials for ancient ceramics, developed by the Shanghai Institute of Ceramics, Chinese Academy of Sciences, were used. In order to ensure the reliability of the chemical analysis, standard reference materials for ancient ceramics, developed by the Shanghai Institute of Ceramics, were used. These standards cover a wide range of ceramic compositions, and their experimental values were obtained using the same EDXRF spectrometer, ensuring comparability. For the SEM and EDS analysis, samples were first etched with 5% hydrofluoric acid (HF) for 30 s to expose the microstructure of the glaze. Following this, the samples were ultrasonically cleaned in distilled water and dried before SEM examination. For EDXRF, samples were left untreated to avoid altering the surface composition.

The standard curves for each element were established using the Delta software (https://www.doc88.com/p-98961477017143.html?s=rel&id=8. Access date: 10 October 2024) [6]. The results are detailed in Table 2. Table 2 provides the chemical compositions of the glazes, where measurement errors were accounted for by performing three repetitions for each element using an Eagle-III energy dispersive X-ray fluorescence (EDXRF, EDAX, Shanghai, China) spectrometer. The standard deviation for each measurement is included in the table, representing the inherent variability of the process. These deviations reflect the heterogeneity of the glaze and slight variations in instrument sensitivity. Before SEM analysis, each specimen was etched with 5% HF for 30 s to remove surface contaminants and expose the underlying microstructure. This process was necessary to reveal the phase separation in the glaze and improve the contrast for SEM imaging.

The colorimetric properties of the glaze surfaces were determined using an NF-333 automatic colorimeter(NDK, Suzhou, China). The instrument was set to a standard light source D65, with a standard observation angle of 10°, and a measuring wavelength range of 400–700 nm at 20 nm intervals, using an 8 mm measuring aperture. Prior to measurement, the colorimeter was calibrated with a reflection calibration board (white and black) provided by the manufacturer. To minimize errors, the color parameters (L*, a*, b*) and tristimulus values (x, y, z) of each sample were measured three times, with the average values recorded as the final color parameters. The results are shown in Table 3.

The size of bubbles on the glaze surface, along with the cross-sectional thickness of the glaze layer, was observed using the VHX-6000 ultra-deep field microscope (manufactured by KEYENCE, Shanghai, China). Additionally, the surface morphology of the glaze layer was examined using an SU-8010 scanning electron microscope (SEM, JEOL Corporation, Shanghai, China). Prior to SEM analysis, each specimen was etched with 5% hydrofluoric acid for 30 s, then cleaned and dried using an ultrasonic cleaner. A Model 550i energy spectrometer (IXRF, Chengdu, China) was employed to analyze the micro-zone composition of the specimens.

## 3. Analysis of Findings and Discussion

### 3.1. Chemical Composition and Formulation of Enamel

Table 2 presents the chemical composition of the opaque glazes from the Changsha Kiln of the Tang Dynasty (Wt %). The SiO_2_ content in these glazes ranges between 62 and 70%, while the Al_2_O_3_ content is relatively low, varying from 7.39% to 12.58%. The glazes also exhibit high concentrations of CaO (12–22%) and MgO (1.28–4.38%), which serve as the primary fluxes in the glaze formulation. By calculating the glaze formulation coefficient b = RO/(RO + R_2_O), as referenced in the relevant literature, it can be determined that the b values for these opaque glazes range from 0.85 to 0.96. ‘R’ refers to the glaze formulation coefficient, specifically the ratio of network-modifying oxides to the total fluxing oxides (R = RO/(RO + R_2_O)). This value helps categorize the glazes into different types. According to the classification criteria for calcium glazes (b > 0.76), calcium-alkali glazes (0.76 < b < 0.50), and alkaline-calcium glazes (b < 0.50), these glazes are classified as calcium glazes [7]. To account for the heterogeneity of the samples, each specimen was analyzed at three different points using SEM-EDS. The variations in Fe_2_O_3_ and CuO contents are presented as mean values with standard deviations in Table 2. This approach helps to mitigate the effect of sample heterogeneity on the reported chemical compositions.

The average MnO content in the opaque glazes is approximately 0.32%, and the P_2_O_5_ content averages around 0.55%. Since plant ash, a common ancient ceramic raw material, typically contains higher levels of MnO and P_2_O_5_ [8], it is likely that a small amount of plant ash was incorporated into the opaque glazes. Further analysis could determine whether additional mineral raw materials, such as limestone or dolomite, rich in calcium and magnesium, were also added to the glaze formulation.

As shown in Figure 1 and Table 1, the opaque glaze specimens exhibit a range of colors; however, the base glazes demonstrate little variation in chemical composition (Table 2). The contents of the coloring oxides, such as Fe_2_O_3_ and CuO, are relatively high but vary significantly between different specimens or within different areas of the same specimen. Specifically, the Fe_2_O_3_ content ranges from 1.20% to 3.65%, while the CuO content varies between 0.01% and 3.01%.

When compared to the green celadon glazes from the Changsha Kiln [9,10], both the celadon and opaque glazes are identified as high-calcium glazes. However, the celadon glazes exhibit slightly lower CaO content and higher Al_2_O_3_ content than the opaque glazes. This suggests that the basic formulation of the opaque glazes differs somewhat from that of the celadon glazes, and that the relatively high silica-to-aluminium ratio in the opaque glazes may contribute to their milky appearance. Additionally, while celadon glazes are primarily colored by iron, the opaque glazes are colored by both iron and copper, resulting in a wider variety of glaze colors.

Previous research indicates that the raw materials for the Changsha Kiln were sourced locally, containing over 70% silicon oxide, which categorizes them as rich silicon oxide materials. The iron content in these materials exceeds 1.5%. Apatite and calcite were used as fluxes in the glaze, while ores such as copper, iron, and manganese served as colorants [11].

### 3.2. Mechanisms of Phase Separation and Coloring

#### 3.2.1. Analysis of Glaze Surface Properties

The experimental specimens from the Changsha Kiln of the Tang Dynasty exhibit a variety of glaze colors, including creamy white, red, blue-green, green, and yellow-black, as illustrated in Figure 1. Notably, even within the same specimen, the glaze can exhibit different coloring effects, which are influenced by the composition of the glaze, firing conditions, and the presence of phase-separated droplets of various sizes. These droplets, combined with small amounts of coloring oxides, contribute to the final appearance of the glazes [12].

The chromaticity parameters of these glazes, as listed in Table 3, provide insight into their color characteristics. For the green glazes, the lightness (L*) values range from 21.21 to 55.97, indicating relatively low brightness. The red-green (a*) values for these glazes range from −11.03 to −2.85, while the yellow-blue (b*) values are positive, showing a blue-green hue when b* values are between 8 and 11, and shifting towards a yellow-green hue as b* values increase above 11.

In contrast, the creamy white glazes exhibit higher lightness values (51.85 to 69.33), with a* values consistently around −3, and b* values ranging from 5.66 to 15.11, indicating a light yellowish tone. Fewer specimens display yellow-black or red glaze colors; each is represented by a single sample. The yellow-black specimen has an L* value of 24.07, an a* value of 6.84, and b* value of 17.52, whereas the red specimen has an L* value of 50.98, an a* value of 9.21, and b* value of 6.86. These data suggests that, except for the red and yellow-black glazes, most opaque glazes from the Changsha Kiln exhibit negative red-green (a*) values and positive yellow-blue (b*) values, indicating an overall yellowish-green hue.

Further analysis of the chromaticity coordinates (x, y) in Table 3 reveals the average values for different glaze colors: green glazes (x = 0.3107, y = 0.3596), yellow-green glazes (x = 0.3493, y = 0.3887), and blue-green glazes (x = 0.3108, y = 0.3613). The creamy white samples exhibit an average x value of 0.3330 and y value of 0.3590, while the red sample has an x value of 0.3539 and y value of 0.3889. The yellow-black sample presents the highest x and y values, at 0.4237 and 0.3967, respectively.

**Figure 2:**
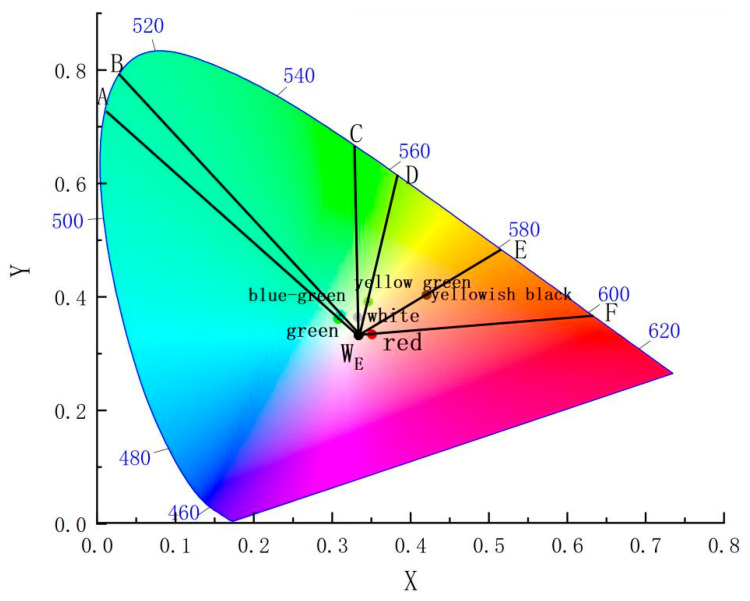
The CIE spatial chromaticity of glazes.

These chromaticity points are mapped in Figure 2 and connected to the equal-energy white point WE (0.3333, 0.3333). The corresponding wavelengths of these points fall within specific spectral ranges: green and blue-green glazes have wavelengths between 500 and 520 nm, the dark green sample approaches 560 nm, and the white sample exceeds 560 nm, which is consistent with the overall green tone of these glazes. The yellow-black sample has a wavelength around 580 nm, within the yellow band of visible light (580–600 nm), while the red sample corresponds to wavelengths around 600 nm, falling within the orange-red spectrum (600–760 nm).

**Figure 3:**
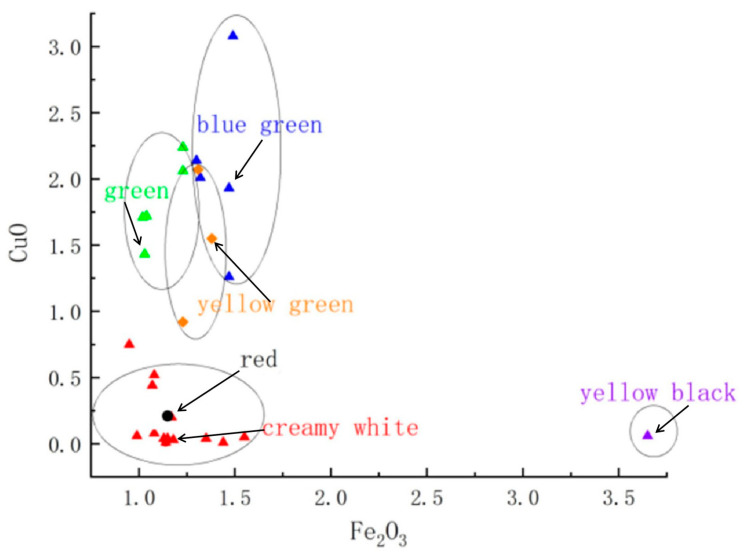
Scatter plot of the relationship between Fe and Cu contents and glaze specimen color.

The chemical analysis of the opaque glazes, as presented in Table 2, indicates that the primary coloring oxides are Fe_2_O_3_ and CuO. Glazes with a yellow-green appearance typically have an average Fe_2_O_3_ content of 1.28% and CuO content of 1.51%. In blue-green glazes, Fe_2_O_3_ ranges from 1 to 1.5%, while CuO ranges from 1.02 to 3.08%. Red glazes have lower contents of these oxides, with Fe_2_O_3_ at 1.15% and CuO at 0.21%. In contrast, the creamy white and yellow-black glazes contain less than 0.52% CuO, with Fe_2_O_3_ content under 2% and 3.65%, respectively. The relationship between these oxide contents and glaze color is visually depicted in Figure 3.

The analysis in this study shows that the interplay between chemical composition, phase separation, and firing conditions directly affects the final color of the opaque glazes. For instance, sample CS-12 displays yellow tones due to its high iron content (3.65% Fe_2_O_3_), while sample CS-14 exhibits a milky appearance due to phase separation. This analysis demonstrates that the final color of the opaque glazes is a result of the complex interplay between the chemical composition, phase separation phenomena, and the specific firing conditions employed during production.

#### 3.2.2. Chemical Coloring Mechanism

In traditional Chinese ceramic glazes, copper (Cu) and iron (Fe) are key transition metal elements responsible for coloring, with copper being second only to iron in significance [13]. Both elements exist as ions within the glass phase of the glaze layer. The valence state of these ions significantly influences the electron layer structure and the position and intensity of absorption bands, which are affected by variations in glaze composition and firing conditions [13]. As a result, the valence electrons selectively absorb specific wavelengths of visible light, producing different colors. Nigel Wood, a renowned British scholar of ancient Chinese ceramics, noted that “copper differs from iron in that it was deliberately added to glazes by Chinese potters as a coloring agent” [14]. To substantiate the claims regarding the oxidation states of Cu and Fe, X-ray photoelectron spectroscopy (XPS) measurements were conducted. The results indicate that Fe exists primarily in the +3 oxidation state, while Cu is present as both Cu^+^ and Cu^2+^, correlating with the observed glaze colors.

Copper can exist in five different states within the glaze: Cu^+^, Cu^2+^, Cu, CuO, and Cu_2_O [15]. The color produced by copper depends on its valence state, which is influenced by the composition of the glaze and the firing atmosphere. The balance between these states determines the final color, and the disappearance of one or more states during firing can result in color changes [16]. The overall glaze color is controlled by the redox balance within the glass matrix [15]. A higher copper content and an oxidizing atmosphere promote the formation of CuO, while higher temperatures lead to the decomposition of CuO [17]. CuO, in its Cu^2+^ form, emits light in the blue-green range of visible wavelengths, resulting in a blue-green hue [18]. High concentrations of copper enhance the patina effect, intensifying the green coloration [15]. In contrast, under reducing conditions, CuO is reduced to Cu^0^, forming colloidal particles that produce a red glaze [18].

Iron (Fe) also plays a critical role in glaze coloration. Being present as iron oxide in the mineral feedstock, in the silicate glass, Fe^2+^ and Fe^3+^ coexist. Fe^2+^ is generally cyan and light green, but it is easily oxidized to Fe^3+^, while Fe^3+^ is easily decomposed to generate Fe^2+^ at high temperature, and Fe^2+^, Fe^3+^, and Fe^3+^ maintain an equilibrium relationship in different proportions under different external field conditions [19]. Fe^3+^, when in tetrahedral coordination, has a strong ability to absorb both ultraviolet and visible light, imparting a yellow-green hue to the glass [20]. High Fe_2_O_3_ content in the glaze, as seen in opaque glazes from the Changsha Kiln, gives the glaze an overall yellowish tint. In an oxidizing atmosphere, iron exists as Fe^3+^, which enhances the yellow tone of the glaze surface. In specimens CS-6, CS-7, and CS-9, the glaze is thinner and contains a higher flux content, causing the glaze to slip during firing and exposing the underlying porcelain, resulting in a yellow appearance. As the Fe_2_O_3_ content increases, so does the Fe^3+^ concentration, leading to a deeper yellow color, as seen in specimens such as CS-12 and CS-3. Consequently, these glazes exhibit a dark yellow-black surface.

### 3.3. Enamel Phase Separation and Physical Coloring Mechanism

#### 3.3.1. Phase Separation Structure

**Figure 4:**
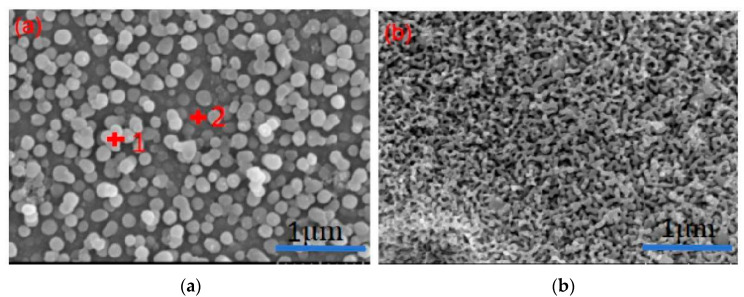
SEM pictures of different parts of the enamel surface of opaque glaze porcelain from the Changsha Kiln. (**a**) At the surface of the glaze layer. (**b**) Near the body of the glaze layer.

Figure 4 shows SEM images of opaque glaze specimens from the Changsha Kiln, dating to the Tang Dynasty. The SEM images of the samples in this experiment are similar, so two SEM images at different positions are selected for explanation. These images reveal a clear liquid–liquid phase separation structure within the glaze, with no evident crystals. The phase separation is more prominent on the glaze surface and becomes less pronounced closer to the body of the specimen. The phase-separated droplets are spherical, vary in size, and have diameters ranging between 50 and 400 nm. Energy dispersive spectroscopy (EDS) analysis, as summarized in Table 4, indicates that the phase-separated droplets are rich in SiO_2_, while the continuous phase is rich in CaO.

#### 3.3.2. Phase Separation Mechanism

**Figure 5:**
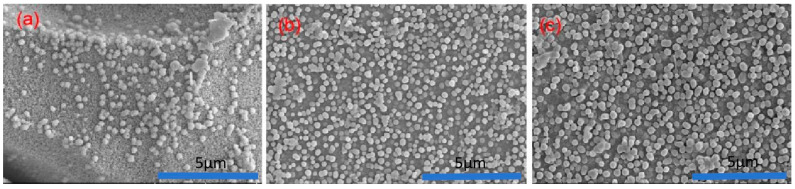
Microscopic images of the effects of different SiO_2_/Al_2_O_3_ molar ratios on phase separation. (**a**) SiO_2_/Al_2_O_3_ = 10.36, (**b**) 11 < SiO_2_/Al_2_O_3_ < 13, (**c**) SiO_2_/Al_2_O_3_ > 13.

The phase separation mechanism is closely related to the SiO_2_/Al_2_O_3_ molar ratio in the glaze, as shown in Figure 5. As the SiO_2_/Al_2_O_3_ ratio increases, significant changes occur in both the size and density of the phase-separated droplets. When the SiO_2_/Al_2_O_3_ ratio is approximately 10.36, the glaze surface begins to exhibit noticeable phase separation. At this ratio, the glaze is primarily green, and the droplets are small and densely distributed. As the SiO_2_/Al_2_O_3_ ratio increases further (between 10.36 and 13), the green color remains dominant, and the phase-separated droplets remain small. However, when the SiO_2_/Al_2_O_3_ ratio exceeds 13, the droplets increase in size, and the glaze color shifts to milky white.

Phase separation differs between the glaze surface and the areas near the body of the specimen, as illustrated in Figure 4. At the surface (labeled a), phase separation is evident, but it diminishes near the body (labeled b). This reduction is primarily due to the higher concentration of Al_2_O_3_ (approximately 20%) in the body, which migrates into the glaze layer during firing. The increased viscosity of the glaze system during cooling inhibits the migration of small droplets, affecting the phase separation process. The SiO_2_/Al_2_O_3_ molar ratio, which influences the [SiO_4_] tetrahedral structure and the linkage of silicon-oxygen groups, also impacts the surface tension and high-temperature viscosity of the glaze melt [21]. A higher Al concentration results in a smaller SiO_2_/Al_2_O_3_ ratio, leading to increased viscosity and reduced mass migration, ultimately inhibiting phase separation.

**Figure 6:**
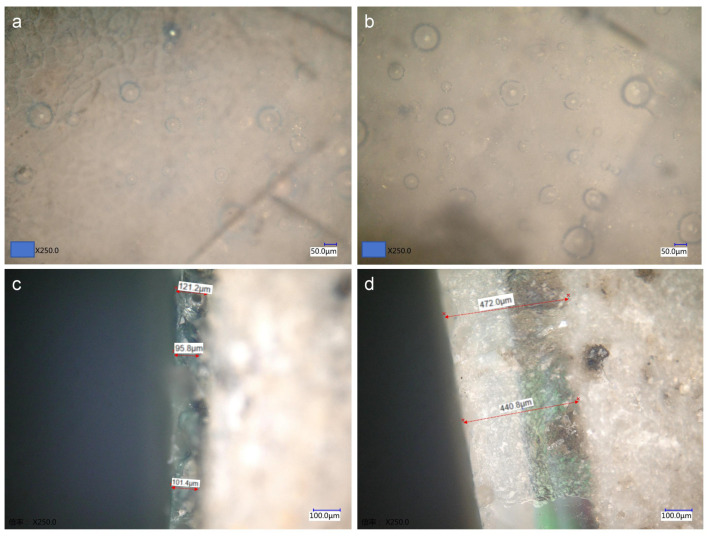
Microscopic image of the thickness of different glaze surfaces and cross-sections (**a**,**c** is CS-9, **b**,**d** is CS-11).

P_2_O_5_ content, which ranges from 0.21% to 0.97% in the glazes, also plays a crucial role in promoting phase separation [22]. A higher P_2_O_5_ content enhances the opacification effect. In addition to phase separation, glaze opacification is also influenced by the presence of bubbles and the thickness of the glaze layer. Microscopic observation of the glaze surface and cross-sections (Figure 6a,b) reveals numerous bubbles of varying sizes, which scatter incident light and contribute to the gas-phase opacification. The thickness of the glaze layer further affects coloring; for example, specimen CS-11, with a glaze layer approximately 450 μm thick (Figure 6c), exhibits a stronger opacification effect compared to specimen CS-9, whose glaze thickness ranges from 90 to 120 μm (Figure 6d).

#### 3.3.3. The Effects of Phase-Separated Droplets on Coloring

**Figure 7:**
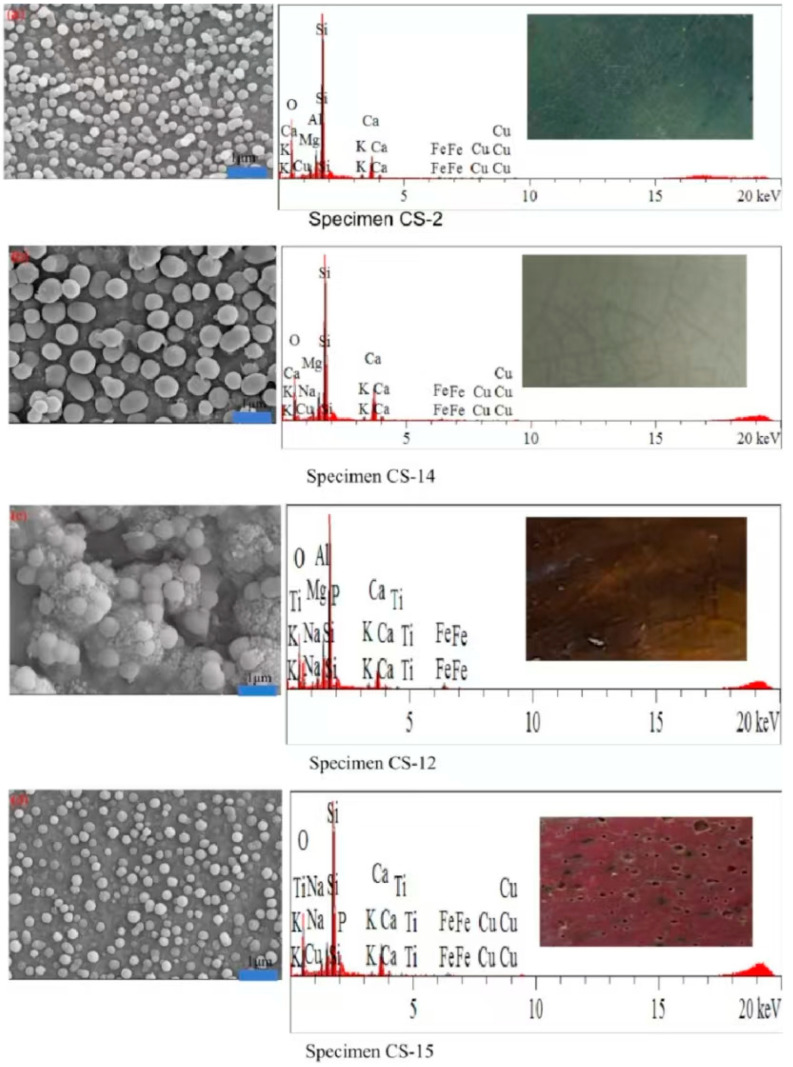
SEM photos and EDS spectra of glaze specimens with different compositions.

The phase-separated structure has a significant influence on the glaze’s coloring. In Figure 7a, the glaze specimen CS-2 shows a phase separation structure on the surface, with droplets ranging in size from 50 to 100 nm. These smaller droplets contribute to dominant Rayleigh scattering, which, upon white light incidence, results in stronger scattering of blue-green light, giving the glaze a light blue-green appearance.

In contrast, the glaze of specimen CS-14 (Figure 7b) contains phase-separated droplets ranging from 80 to 400 nm, which are more consistent with Mie scattering, resulting in a white glaze [12]. Meanwhile, the specimens CS-12 and CS-15 (Figure 7c,d) display evident phase separation structures but exhibit yellow, yellow-black, and red colors, suggesting that the glaze’s chemical composition strongly influences the final color, in addition to the physical phase separation.

## 4. Conclusions

### 4.1. High-Calcium Opaque Glazes from the Changsha Kiln

The opaque glazes from the Changsha Kiln are primarily high-calcium glazes. They contain small amounts of manganese oxide (MnO) and phosphorus pentoxide (P_2_O_5_). The high concentrations of coloring oxides, such as iron (Fe) and copper (Cu), suggest that plant ash and copper-rich minerals were likely used as colorants during the glaze preparation process.

### 4.2. Liquid–Liquid Phase Separation Structure

Microscopic analysis reveals a distinct liquid–liquid phase separation structure in the opaque glazes. This structure is more prominent near the surface and gradually diminishes towards the body of the specimen. The SiO_2_/Al_2_O_3_ molar ratio and P_2_O_5_ content in the glaze play a significant role in the phase separation process. The phase-separated droplets are rich in SiO_2_, while the continuous phase is dominated by CaO. In addition to the size of the droplets, the presence of bubbles and the thickness of the glaze layer also contribute to the opacification effect observed in these glazes.

### 4.3. Glaze Coloring Mechanism

The color of the glaze is influenced by both the composition of the coloring oxides and the size of the phase-separated droplets. A high Fe_2_O_3_ content gives the glaze a yellow hue. Under oxidizing conditions, the CuO content increases, resulting in an overall green color. When the size of the phase-separated droplets is less than 100 nm, Rayleigh scattering occurs, causing the glaze surface to appear blue-green. Conversely, when the droplets are larger, Mie scattering dominates, producing a milky white glaze. In a reducing atmosphere, CuO transforms into Cu0 in colloidal form, which imparts a red color to the glaze.

## Figures and Tables

**Table 1:** Appearance characteristics of milky turbid-glazed porcelain specimens from Changsha Kiln.

Specimen No.	Body	Glaze
CS-1	Fine and yellow body; dense; 5.5 mm in thickness	Green glaze with bluish tinge; lustreless surface; 188.2 μm in thickness
CS-2	Greyish white body; rough and porous; 4.8 mm in thickness	Green glaze with bluish tinge; yellowish at the mouth rim; lustreless surface and fine craquelure; 310.7 μm in thickness
CS-3	Greyish white body; fine texture; 5 mm in thickness	Green glaze with a yellowish tinge; lustreless surface; glaze surface peeling (glaze not well bonded to the body); 286.3 μm in thickness
CS-4	Yellow body; rough, with a lot of bubbles; 3 mm in thickness	Extensive green glaze tinged with white glaze; overall yellowish; craquelure on glaze surface; lustreless; 406.3 μm in thickness
CS-5	Grey body; fine texture; 6 mm in thickness	Interspersed green and white glazes; overall yellow; lustreless glaze surface; 406.3 μm in thickness
CS-6	Yellow body; loose, with a lot of bubbles; 5.5 mm in thickness	Creamy white glaze; rhyolite mixed with green glaze; blue-toned, lustrous glaze surface; 265.3 μm in thickness
CS-7	Grey body; rough, with many bubbles; 5.6 mm in thickness	Green glaze mixed with white glaze; bluish tinge, with a lustreless surface; raised; 365.1 μm in thickness
CS-8	Grey body; rough, with bubbles; 5 mm in thickness	Green glaze mixed with white glaze, with a lustreless surface; 200.5 μm in thickness
CS-9	Grey body; rough, with many bubbles; 5.5 mm in thickness	Green glaze with white glaze; bluish tinge, with areas of black spots; low lustre; 227.9 μm in thickness
CS-10	Grey body; rough, with bubbles; 5.5 mm in thickness	Yellow-green glaze with rhyolite creamy white glaze; lustrous; 352.9 μm in thickness
CS-11	Yellow body; rough, with bubbles; 5 mm in thickness	Yellow-green glaze mixed with creamy white glaze, with craquelure on the glaze surface; 196.8 μm in thickness
CS-12	Yellow body; dense; small bubbles; 7 mm in thickness	Interspersed yellow and black glaze, white glaze, and blue-green glaze; overall yellowish; lustrous glaze surface; 390.6 μm in thickness
CS-13	Greyish white body; fine texture, with few bubbles; 4.5 mm in thickness	Creamy white glaze with a yellowish tinge; yellowish; lustrous, with fine craquelure on glaze surface; 225.1 μm in thickness
CS-14	Yellow body; rough, with bubbles; 5 mm in thickness	Creamy white glaze with a yellowish tinge; rhyolitic; lustrous; 395.1 μm in thickness
CS-15	Yellow body; fine texture; 4 mm in thickness	Red glaze with a hint of white; lustrous glaze surface; open flakes; numerous bubbles; 383.7 μm in thickness
CS-16	Grey body; rough, with bubbles; 4 mm in thickness	Creamy white glaze; yellowish, with craquelure on glaze surface; low lustre; 552.5 μm in thickness

**Table 2:** Chemical composition of opaque glazes from the Changsha Kiln.

No.	Glaze Layer Color	Wt %											SiO_2_/Al_2_O_3_ Molar Ratios
		Na_2_O	MgO	Al_2_O_3_	SiO_2_	K_2_O	CaO	TiO_2_	Fe_2_O_3_	MnO	CuO	P_2_O_5_	
CS-1	Green	0.03	4.26	8.32	61.84	2.01	21.17	0.36	1.02	0.41	1.71	0.69	12.61
CS-2	Green	0.31	3.37	8.14	63.15	1.84	20.60	0.37	1.23	0.39	2.06	0.52	13.16
CS-3	Green	0.45	3.65	8.36	63.29	2.77	19.08	0.36	1.04	0.34	1.72	0.66	12.85
CS-4-1	Creamy white	0.03	3.33	7.35	64.32	2.42	20.17	0.31	1.08	0.36	0.52	0.62	14.85
CS-4-2	Blue green	0.29	2.93	9.21	63.70	2.94	18.22	0.38	1.32	0.31	2.01	0.56	11.74
CS-4-3	Yellow green	0.03	2.90	9.49	63.84	2.82	18.20	0.40	1.31	0.29	2.07	0.55	11.41
CS-5-1	Creamy white	0.03	3.25	8.39	64.27	1.88	19.69	0.37	1.13	0.35	0.04	0.56	13.00
CS-5-2	Blue green	0.80	3.18	9.45	63.56	2.43	17.68	0.43	1.47	0.28	1.26	0.52	11.41
CS-6-1	Creamy white	1.05	3.30	8.13	62.44	2.19	20.38	0.34	1.17	0.29	0.20	0.61	13.03
CS-6-2	Green	0.03	3.09	8.34	62.80	2.44	20.94	0.33	1.03	0.30	1.43	0.65	12.78
CS-7-1	Blue green	1.95	2.18	8.63	64.79	2.22	17.31	0.43	1.49	0.26	3.08	0.54	12.74
CS-7-2	Creamy white	1.46	1.78	7.94	67.33	2.07	16.96	0.37	1.08	0.27	0.08	0.45	14.39
CS-8-1	Creamy white	0.29	1.28	9.18	70.82	3.55	12.24	0.28	1.35	0.22	0.04	0.18	13.09
CS-8-2	Green	0.03	2.83	8.40	64.65	2.53	18.93	0.38	1.23	0.27	2.24	0.60	13.06
CS-9-1	Creamy white	1.86	2.91	7.40	63.75	1.63	19.98	0.28	1.18	0.24	0.03	0.66	14.62
CS-9-2	Blue green	0.45	2.63	9.40	66.50	2.06	16.14	0.35	1.47	0.19	1.93	0.52	12.00
CS-10-1	Yellow green	0.53	3.13	10.23	63.02	2.32	17.98	0.41	1.38	0.25	1.55	0.51	10.45
CS-10-2	Dark green	0.27	3.13	9.12	62.62	1.89	20.28	0.38	1.30	0.27	2.14	0.50	11.65
CS-10-3	Creamy white	1.31	2.80	7.53	62.37	1.72	21.90	0.31	1.07	0.29	0.44	0.61	14.05
CS-11-1	Creamy white	1.07	3.53	7.93	64.62	1.98	18.37	0.35	1.15	0.40	0.04	0.53	13.83
CS-11-2	Yellow green	0.03	3.70	9.94	63.08	2.37	18.25	0.39	1.23	0.36	0.92	0.58	10.77
CS-12-1	Blue green	0.03	3.77	8.27	65.06	1.91	18.69	0.33	0.95	0.34	0.75	0.58	13.35
CS-12-2	Creamy white	0.37	3.61	8.20	66.70	2.05	16.21	0.31	1.55	0.37	0.05	0.47	13.80
CS-12-3	Yellow black	0.31	2.93	10.32	63.02	2.36	16.01	0.40	3.65	0.35	0.06	0.48	10.36
CS-13	Creamy white	0.03	2.87	8.29	65.97	2.92	17.43	0.34	1.14	0.24	0.01	0.36	13.50
CS-14	Creamy white	0.03	3.81	8.10	64.34	1.98	19.27	0.34	1.14	0.34	0.01	0.70	13.48
CS-15-1	Creamy white	0.61	3.60	7.39	61.80	2.04	22.23	0.34	0.99	0.40	0.06	0.62	14.19
CS-15-2	Red	0.03	3.13	7.98	62.23	2.04	22.10	0.34	1.15	0.39	0.21	0.62	13.23
CS-16	Creamy white	0.40	3.54	8.59	63.83	2.72	18.14	0.35	1.44	0.38	0.01	0.56	12.61
Average		0.49	3.12	8.55	64.13	2.28	18.78	0.36	1.30	0.32	0.92	0.55	
Standard deviation		0.56	0.61	0.82	1.92	0.43	2.16	0.04	0.48	0.06	0.94	0.10	

**Table 3:** L*, a*, b* values of different enamels.

No.	L*	a*	b*
CS-1	39.16	−8.89	2.4
CS-2	34.89	−8.35	0.78
CS-3	47.86	−10.15	8.81
CS-4-1	62.00	−1.25	15.11
CS-4-2	52.34	−9.89	2.56
CS-4-3	48.86	−9.65	11.29
CS-5-1	61.99	−1.69	13.04
CS-5-2	52.56	−6.15	9.45
CS-6-1	58.14	−0.96	6.69
CS-6-2	32.71	−7.18	0.61
CS-7-1	41.16	−9.27	4.16
CS-7-2	68.32	−3.15	10.45
CS-8-1	67.34	−2.14	12.53
CS-8-2	49.75	−11.03	10.14
CS-9-1	49.8	−1.23	8.66
CS-9-2	42.66	−4.50	8.99
CS-10-1	35.6	−4.44	14.11
CS-10-2	21.21	−3.79	8.05
CS-10-3	60.63	−2.06	9.87
CS-11-1	57.16	−1.37	7.46
CS-11-2	38.43	−2.85	14.96
CS-12-1	55.97	−8.71	9.36
CS-12-2	66.85	−3.3	14.42
CS-12-3	24.07	6.84	17.52
CS-13	51.85	−3.44	7.28
CS-14	69.33	−1.73	14.02
CS-15-1	61.41	−5.56	5.66
CS-15-2	50.98	9.21	6.86
CS-16	42.93	−5.12	8.52

**Table 4:** EDS data of specimens in Figure 4.

	No.		O	Na	Mg	Al	Si	K	Ca	Ti	Fe	Cu
1-phase-separated droplet	16	Average	39.29	1.44	3.24	6.65	34.24	1.29	7.59	1.13	2.06	3.05
		Standard deviation	7.66	0.44	0.40	0.47	4.16	0.26	1.93	0.35	0.65	2.04
2-Continuous phase	16	Average	41.36	1.34	3.33	6.34	30.58	1.33	9.73	1.19	1.75	3.04
		Standard deviation	9.07	0.17	0.36	0.49	3.33	0.46	2.79	0.46	0.85	2.28

## Data Availability

The original contributions presented in the study are included in the article, further inquiries can be directed to the corresponding author.
